# 

**Published:** 1850-01

**Authors:** 


### CONTENTS

OF THE

### MEDICAL EXAMINER.

NEW SERIES—NO. LXI.—JANUARY, 1850.

### ORIGINAL COMMUNICATIONS.

A Case of the Cheiloplastic Operation, with two portraits. By Abraham Stout, M. D., of Easton, Pennsylvania, - - - - - 13

A Modified Operation in Excision of the Os Maxillare Superius. By W. E. Horner, M. D., Professor of Anatomy in the University of Pennsylvania, and Senior Surgeon in the St. Joseph's Hospital, - - - - - - - - - - - - - - 16

Case of Fracture of both Clavicles. By Wm. Quail, M. D., of Pittsburg. (Communicated by Prof. Mütter,) - - - - - 19

Rupture of the Spleen. Autopsy. By M. G. Whitney, M. D., of Kingston, Pennsylvania, - - - - - - - - . 20

Pennsylvania Hospital.—Surgical Wards.—Service of Dr. Norris, - 21

### BIBLIOGRAPHICAL NOTICES.

On the Diseases of Infants and Children. By Fleetwood Churchill, M. D., M. R. I. A., Hon. Fellow of the College of Physicans, Ireland; Hon. Member of the Philadelphia Medical Society, &c., &c., - - - - - - - - - - - - - - 26

A Treatise on the Diseases of the Bones. By Edward Stanley, F. R. S., President of the Royal College of Surgeons, England, and Surgeon to St. Bartholomews' Hospital, - - - - - - 30

Principles of Human Physiology, with their chief applications to Pathology, Hygiene and Forensic Medicine. By William B. Carpenter, M. D., F. R. S., F. G. S., &c., &c., - - - - - 35

An Essay on Intestinal Auscultation. By Charles Hooker, M. D., Professor of Anatomy and Physiology in Yale Medical College, 37

Surgical Anatomy. By John Maclise, Surgeon, - - - - - 40

Contributions to Physiology. By Bennet Dowler, M. D., of New Orleans, - - - - - - - - - - - - - - 42

### EDITORIAL.

Changes and appointments, - - - - - - - - - 51

### RECORD OF MEDICAL SCIENCE.

### ANATOMY AND PHYSIOLOGY.

Experimental Inquiry into the Causes of the Sounds of the Heart, Confirmatory of the Views of Dr. Billing. By Harris C. Brakyn, Esq., (with cuts,) - - - - - - - - - 53

Observations on the Occipital and Superior Maxillary Bones of the African Cranium, by John Neill, M. D., - - - - - 57

### PATHOLOGY AND PRACTICE OF MEDICINE.

On the Neck as a Medical Region; in Syncopal Seizure, &c. By Marshall Hall, M. D., F. R. S., &c., - - - - - - 57

### MATERIA MEDICA AND THERAPEUTICS.

On Nux Vomica in Impotence and Spermatorrhœa. By M. Duclos, 62

On Valerian in Epilepsy. By M. Chauffard, - - - - - 63

On the Treatment of Chilblains. By M. Ossieur, - - - - - 64

On the Pharmaceutical and Toxicological Applications of Carbon. By M. Esprit, - - - - - - - - - - - - - - 64

Chocolate and Broma, - - - - - - - - - - - - - 65

SURGERY.

Gangrene of the Scrotum—Autoplasty—Use of Collodion, - - 66

Unsuccessful Application of a Ligature on the Femoral Artery for a Wound of the Anterior Tibial, followed by Amputation. By W. S. Davis, Esq., M. R. C. S., &c., Heytesbury, - - - 67

CHEMISTRY.

Fraudulent Uses of Sulphuric Acid. By H. Letheby, M. B., London, Lecturer on Chemistry at the Medical College of the London Hospital. - - - - - - - - - - - - 70

OBSTETRICS.

Vicarious Menstruation, - - - - - - - - - - - 74

